# Lady Warwick's Home for Crippled Children

**Published:** 1896-06-06

**Authors:** 


### LADY WARWICK'S HOME FOR CRIPPLED CHILDREN.

This Home is situated at Milverton, Warwick, and has been founded by the Countess of Warwick, who has purchased an excellent house, with ample grounds, and has furnished and fitted up the Home upon the most approved principles. Having made a personal inspection, we are happy to be able to state that it is beyond doubt the best organised and arranged institution of the kind in the country. Everything connected with it has been carefully thought out. The dormitories are airy, with polished floors, most comfortable bedding, and suitable lockers. Pictures and toys abound, and, having regard to the fact that the buildings consist of an old dwelling-house which has been adapted to the purposes of a hospital, we consider them excellent.

In selecting her nurses Lady Warwick has exercised a wise choice. The sister-in-charge is genuinely interested in her work, and her nurses and probationers are young women who are evidently fond of children, and who delight to take trouble to make their little charges happy and comfortable. When we visited the Home, the whole of the children were having tea in the grounds under the trees, and we had a good opportunity of observing how they were cared for and their general condition. A happier, merrier, cleaner, or brighter party it would be difficult to imagine, and the excellent state of the children proved the constant care and attention which they receive at the hands of the nursing staff. The condition of these children was in marked contrast to that observed at certain other homes which shall be nameless, and proved the excellent influence which Lady Warwick exercises over all those who work in public matters under her direction.

The rules have been well thought out, and are as few in number and as simple as possible. They provide for the admission of children selected by the authorities of orthopædic and other hospitals, the Invalid Children's Aid Association, and similar institutions. The beds are free to the different institutions, provided the children's railway expenses are paid and that proper clothing is sent with them; all other expenses are borne by the Home. The Home provides accommodation for seventeen children. None must exceed the age of fourteen or be less than two years old. There is no limit to the time they may remain, and many do so for months and even years. Indeed, the chief object of the Home is to restore the little ones to health before being sent home to less favourable circumstances. This renders it an exceptionally valuable institution, as there are few which welcome these lengthy cases; and that it has supplied a want is evidenced by the fact that the present inmates have been drawn from London, Birmingham, and other great centres of population, as well as from Warwick, and the adjacent counties; and the Home has always been full since its establishment. No visitor to Leamington can fail to be impressed by the wide popularity of the Countess of Warwick, whose untiring labours for others in so many directions is highly valued by all classes of the people to whose interests she devotes so large a share of her time.

